# Determining factors that maintain physical function or increase frailty using the Kihon checklist among community-dwelling older adults: a six-year longitudinal study in Agano, Japan

**DOI:** 10.1186/s12877-023-04055-1

**Published:** 2023-05-30

**Authors:** Norio Imai, Takuya Yoda, Yoji Horigome, Reiko Murakami, Masashi Wakasugi, Toshihide Fujii, Masayuki Ohashi, Hiroyuki Kawashima

**Affiliations:** 1 Division of Comprehensive Musculoskeletal Medicine, Niigata University Graduate School of Medical and Dental Sciences, Niigata City, Japan; 2 Department of Orthopedic Surgery, Agano City Hospital, Agano City, Japan; 3 Division of Orthopedic Surgery, Department of Regenerative and Transplant Medicine, Niigata University Graduate School of Medical and Dental Sciences, Niigata City, Japan

**Keywords:** Frailty, Kihon Checklist, Japan, Important factor

## Abstract

**Background:**

A significant increase in the older adult population in Japan will significantly increase healthcare costs. This study aimed to examine the risk factors contributing to robustness transitioning to frailty in older residents.

**Methods:**

Participants were aged 70 in 2016 and 76 in 2022. Participants were evaluated using the Kihon Checklist (KCL).

**Results:**

Participants for this longitudinal study included 444 older persons who completed the KCL surveys in 2016 and 2022. The follow-up rate was 80.6%; therefore, 358 participants were included in the analysis. The median KCL score increased significantly from 2 to 2016 to 3 in 2022 (*p* < 0.001). The prevalence of robustness significantly decreased from 60.9 to 48.6% (*p* = 0.042). In a stepwise logistic regression analysis, robustness was independently associated with regular continuous walks for 15 min and a body mass index of above 18.5%. The following variables were associated with the transition to prefrailty: experiencing a fall in the past year and not going out at least once a week. For the transition to frailty, the variables were turned to family or friends for advice, experienced a fall in the past year, and felt helpless in the last two weeks. The independent factor for the transition from prefrailty to frailty was having a BMI of less than 18.5. In contrast, the independent factor for improving from frailty to robustness or prefrailty was going out at least once a week.

**Conclusions:**

We recommend maintaining continuous walking for more than 15 min, maintaining a BMI of at least 18.5, and going out more than once a week to improve being house-bounded and depressive mood, not only to prevent the transition to prefrailty or frailty but also to improve frailty.

**Supplementary Information:**

The online version contains supplementary material available at 10.1186/s12877-023-04055-1.

## Background

The older adult population is proliferating in Japan. The percentage of people over 65 in 2020 was 28.8% and is expected to increase to 30% by 2025 and 37.7% by 2050 [1]. This increase in the older adult population will significantly increase healthcare costs [2]. In preparation for this aging society, the Japanese government promotes healthy aging to facilitate the maintenance of physical functions and prevent disability and dependence [3]. They have identified frailty as one of their targets.

Frailty is a common old-age syndrome and refers to increased vulnerability to stressors and outcomes, such as disability, need for care, and death [4]. Frailty also includes psychological and social factors [4]. The frailty phenotype described by Fried et al. uses data from the Cardiovascular Health Study and includes unintentional weight loss (10 lbs in the past year), self-reported exhaustion, weakness (grip strength), slow walking speed, and low physical activity [5], which is one of the most widely used definitions of frailty. In Japan, the Japanese version of the Cardiovascular Health Study (J-CHS) criteria, which modifies and adapts Fried et al.’s criteria for body-weight loss, exhaustion, grip power, walking speed, and physical inactivity to the Japanese population, is widely used [6]. In contrast, Mitnitski et al. presented a cumulative deficit model of frailty that considers the physical component and psychosocial facets [7].

The Kihon Checklist (KCL) is a widely used self-assessment tool that measures dependency risk among community-dwelling older adults (Table S1 in Additional File 1) [8, 9]. The KCL consists of 25 items divided into seven categories: activities of daily living (ADL), physical activity, nutritional status, oral function, house restraint, cognitive status, and depressive mood. Based on Fried et al.’s frailty criteria, the KCL shows excellent validity in assessing frailty [10, 11] and in predicting the incidence of dependence and death within 3 years [12]. Moreover, it takes approximately 15 min to complete the questionnaire [13]; therefore, KCL is a convenient tool for evaluating physical status. It may be, however, that the importance of each question may differ, and identifying the important questions influencing the maintenance of robustness or the transition to frailty would be beneficial, although higher KCL scores have been well established as risk factors for needing assistance and care [11].

Therefore, identifying the primary factors that contribute to these conditions makes it easier for physicians and governments to intervene. This study aimed to analyze the risk factors for the transition to frailty among 25 items of KCL over 6 years from 2016 to 2022. Based on the items of the KCL, we hypothesized that multiple independent factors contribute to the maintenance of robustness or preventing frailty.

## Methods

**Fig. 1:**
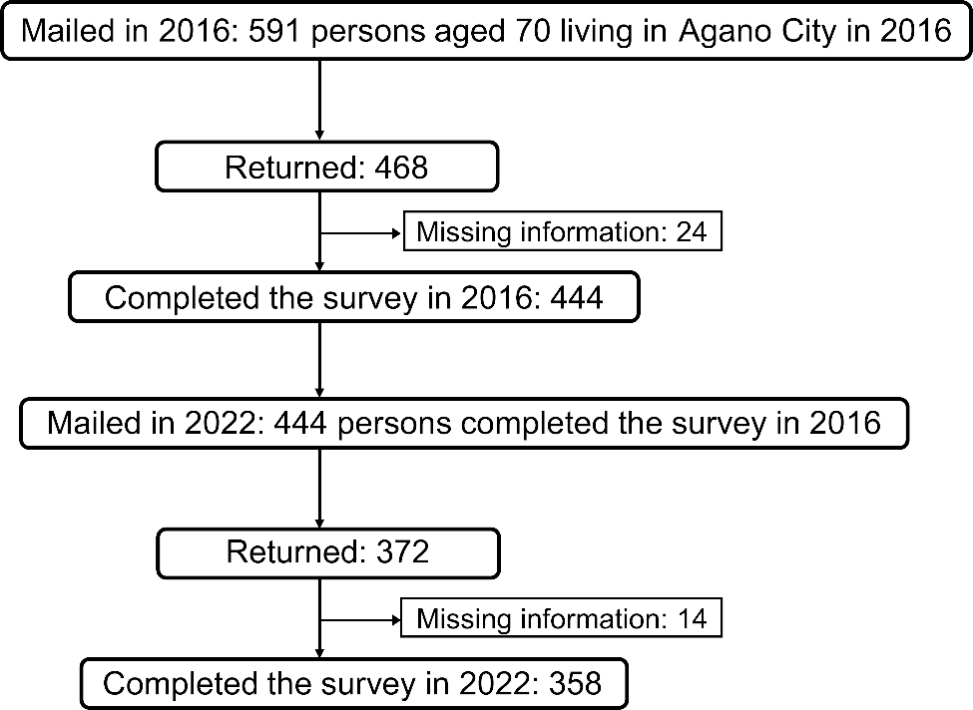
Flow diagram of the participant-selection process

Located in northeastern Niigata Prefecture, Agano City’s local government has been sending the KCL survey via mail annually since 2011. In the current study, data from these surveys were retroactively obtained from the government for residents of Agano City who were 70 years old since healthy life expectancy in Japan is 72 years for men and 75 years for women [14]. In 2016, Agano City had 591 persons aged 70 (302 women and 289 men) and 476 persons returned the survey form. After those with missing information were excluded, 444 potential participants remained. Of these 444 participants, 372 persons returned the questionnaire. After those with missing information were excluded, 358 completed the project in 2022 (Fig. 1). Therefore, the participants of this longitudinal survey included 444 older persons aged 70 years as of 2016 (follow-up rate: 80.6%) who completed the KCL survey in both 2016 and 2022.

### The Kihon Checklist (KCL)

KCL is a self-report questionnaire including 25 “yes” and “no” items: 5 of ADL, 5 of physical activity, 2 of nutritional status, 3 of oral function, 2 of house boundedness, 3 of cognitive status, and 5 of depressive mood items (Table S1 in Additional File 1) [9]. Each question received a score of 0 to 1, with the minimum score possible on the survey being 0 and the maximum being 25. Scores of 0–3 were defined as *robustness*, 4–7 as *prefrailty*, and 8 or higher as *frailty* [11], with higher KCL scores indicating a higher risk of needing assistance and care, as previously reported [11]. To measure frailty status, improved, unchanged, or worsened transitions in physical function were determined by changes in KCL scores from 2016 to 2022. A decrease in total or dimensional KCL scores in 2022 compared to 2016 would indicate improving transitions, whereas an increase in score indicates worsening transitions. The 6-year frailty incidence rate was calculated by comparing the number of older adults who were robust or pre-frail in 2016 to those who were frail in 2022.

### Statistical analysis

SPSS software (version 28; IBM Corp., Armonk, NY, USA) was used for all statistical analyses. Continuous values are expressed as mean ± standard deviation or median (interquartile range [IQR]). In addition, for each of the 25 items of the KCL in 2016, stepwise logistic regression was used to identify independent risk factors for: (1) the maintenance of robustness, using participants who were identified as being robust in 2016; (2) the transition from robustness to prefrailty, using participants who were evaluated as robust in 2016 and as prefrail in 2022; (3) the transition from robustness to frailty, using participants who were identified as robust in 2016 and as prefrail in 2022; and (4) the improvement from frailty to robustness or prefrailty in 2022, using participants evaluated as being frail in 2016 and as either prefrail or robust in 2022. Factors with *p* < 0.20 in univariate analysis were included in the multivariate logistic regression using a stepwise method; a value of *p* < 0.05 was considered statistically significant. In the residual analysis, if the absolute value of the adjusted residuals was greater than 1.96, it was considered significantly different from the expected frequency at *p* < 0.05.

### Ethics statement

The Institutional Review Board of Niigata University reviewed and approved this study (2019–0221). We conducted all methods of this study according to relevant guidelines and regulations. The requirement for informed consent was waived because this was a retrospective study without any interventions.

## Results

**Fig. 2:**
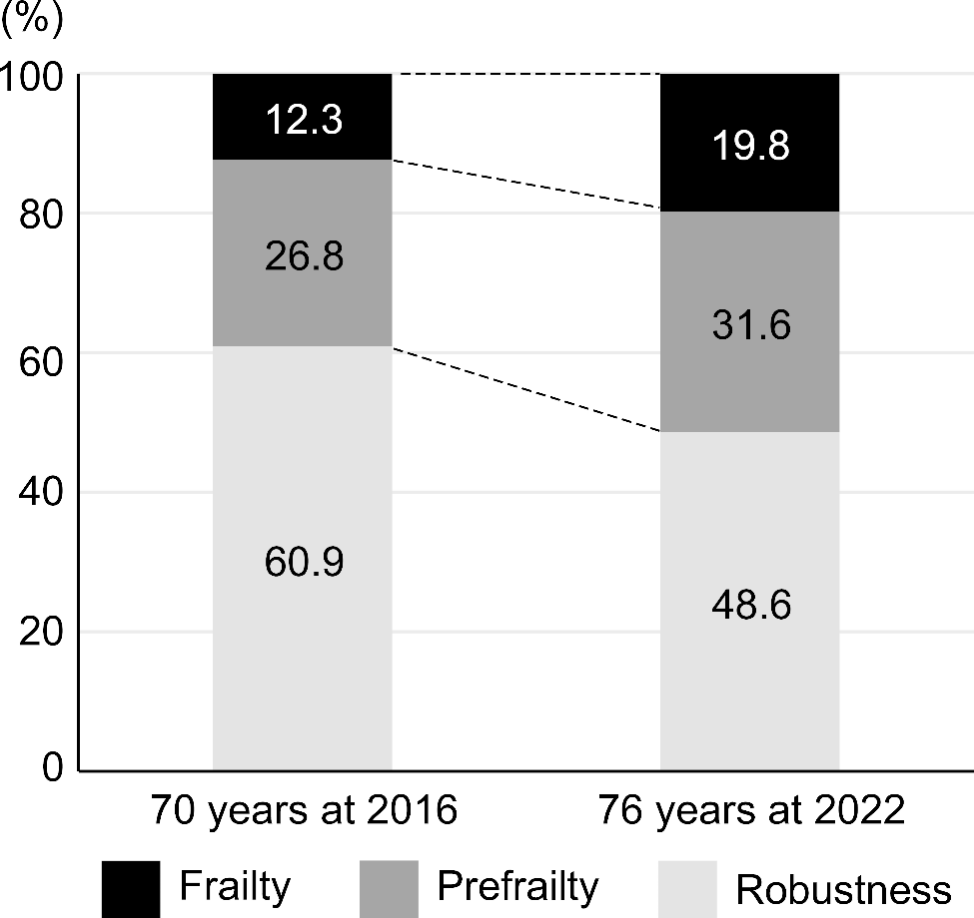
Frailty, prefrailty, and robustness rates 2016–2022. Robustness significantly decreased, frailty and prefrailty were unchanged

The participants in this study were 358 older persons, including 185 women (51.7%) and 173 men aged 70 years in 2016 and 76 years in 2022. The median value of KCL significantly increased from 2 (IQR, 1–4) in 2016 to 3 (IQR, 1–6) in 2022 (*p* < 0.001; Table 1). The rates of prefrailty and frailty were significantly increased from 12.3% (n = 44) to 19.8% (n = 71; adjusted residual, 1.488) and from 26.8% (n = 96) to 31.6% (n = 113; adjusted residual, 1.020), respectively. However, this result was not significant. The rate of robustness was significantly decreased from 60.9% (n = 218) to 48.6% (n = 174; adjusted residual, -3.304; chi-square test, *p* = 0.042; Fig. 2).

**Table 1:** Change of the KCL score and each dimensions from 2016 to 2022

	Total	ADL	Physical activity	Nutritional status	Oral function	House-boundedness	Cognitive status	Depressive mood
IQR 2016	2 (1–3)	0 (0–1)	0 (0–1)	0 (0–0)	0 (0–1)	0 (0–0)	0 (0–0)	0 (0–0)
IQR 2022	3 (1–6)	0 (0–1)	0 (0–2)	0 (0–1)	0 (0–1)	0 (0–1)	0 (0–1)	0 (0–1)
*p*-value	< 0.001	< 0.001	< 0.001	0.094	< 0.001	< 0.001	0.001	< 0.001

ADL: activities of daily living; KCL: Kihon checklist

Regarding the seven dimensions of the KCL: ADL, physical activity, nutritional status, oral function, house restraints, and depressed mood deteriorated (*p* < 0.001), while cognitive status remained unchanged from 2016 to 2022 (Table 2).

**Table 2:** The rate of robustness, prefrailty, and frailty in 2016 and 2022

		Status 6-years follow-up (2022)		
		Robust	Prefrailty	Frailty
Baseline status (2016)	Robust	146 (40.8%)	52 (14.5%)	20 (5.6%)
	Prefrailty	22 (6.1%)	49 (13.7%)	25 (7.0%)
	Frailty	6 (1.7%)	12 (3.4%)	26 (7.2%)

Upper low: number of the participants, lower low: percentage

The robustness of the majority of the participants (40.8%) remained unchanged (Table 2). Among those who were robust at baseline, 33.0% showed a decline in their frailty status, of which 23.8% were prefrail, and 9.2% were frail (Table 2). The percentage of older adults evaluated as prefrail at baseline who transitioned to robust after 6 years was 22.9%, and the percentage that transitioned to frail was 26.0% (Table 2). Moreover, approximately 40% of those who were evaluated as frail at baseline improved to prefrailty (27.3%) or robustness (13.6%; Table 2).

Stepwise logistic regression analysis showed that the questions that determined independent factors for maintaining robustness were “Q8. Do you normally walk continuously for 15 minutes?” and “Q12. Do you have a BMI of at least 18.5?” The questions for transitions from robustness to prefrailty were “Q9. Have you fallen in the past year?” and “Q16. Do you go outside at least once per week?” For the transition from robustness to frailty, the questions were “Q5. Do you consult your family or friends?” “Q9. Have you fallen in the past year?” and “Q24. Have you felt helpless in the past two weeks?” (Table 3). An independent factor for the transition from prefrailty to frailty was “Q12. Do you have a BMI of at least 18.5?” (Table 3). Finally, the independent factor for improvement from frailty to robust or prefrailty was “Q16. Do you go out at least once a week?” (Table 3).

**Table 3:** The results of logistic regression

	Factor	Answer	Odds ratio	95% confidential interval	*p*-value
Maintain robustness	Q8. Do you normally walk continuously for 15 min?	Yes	5.096	1.255–20.687	0.023
	Q12. BMI is less than 18.5	No	4.185	1.481–11.824	0.007
Transition robustness to prefrail	Q9. Have you experienced a fall in the past year?	Yes	4.149	1.140-15.152	0.031
	Q16. Do you go out at least once a week?	No	3.551	1.241–9.324	0.005
Transition robustness to frail	Q5. Do you turn to your family or friends for advice?	No	5.049	1.250-19.227	0.010
	Q9. Have you experienced a fall in the past year?	Yes	2.852	1.054–7.715	0.039
	Q24. In the last two weeks have you felt helpless?	No	4.119	1.121–14.551	0.017
Transition prefrailty to frailty	Q12. BMI is less than 18.5	Yes	1.240	1.012–1.520	0.038
Improve frail to robustness or prefrail	Q16. Do you go out at least once a week?	Yes	4.902	1.214–18.823	0.037

## Discussion

Frailty status evaluated with the KCL showed that among older residents aged 70 in 2016 and 76 in 2022, 60.9% and 48.6% were robust, 26.8% and 31.6% were prefrailty, 12.3% and 19.8% were frailty in 2016 and 2022, respectively. The prevalence of frailty among older Japanese adults ranged from 4 to 17.2% among adults over age 65 years [12, 15, 16], including community-dwelling adults who were approximately 70 years of age [17], similar to the baseline year of this study (i.e., 2016).

Recently, KCL has been proposed as a tool for predicting long-term risk of care [11], healthy life expectancy [18], and cognitive function [19]. Therefore, in aging countries like Japan, KCL surveys are valuable for identifying and assessing frailty status at a low cost since they are conducted by mail or other self-reporting methods. Furthermore, understanding the frailty process and predicting how the frailty status of an older person will change is essential for designing effective interventions to prevent or delay its progression.

The present study found a significant increase in total KCL scores from baseline to the 6-year follow-up. Moreover, the prevalence of frailty, as measured by KCL, increased from 12.3 to 19.8%, but the results were not significant. These findings are included in previous studies [16, 20, 21] that reported older age as an important risk factor for frailty status. However, there are few findings on the especially important factors in maintaining a healthy status regarding KCL survey items.

In the current study, factors that were found to maintain robustness were continuous walking for 15 min and a BMI of at least 18.5. As walking ability is closely related to locomotion, resting state, range of motion, and gait stability [22, 23], it was strongly associated with the maintenance of robustness. In addition, those who were underweight at midlife had a higher risk of becoming frail eight years later [24]. Therefore, these factors may be the most important for maintaining robustness. However, studies have reported compelling data regarding the adverse effects of obesity and high waist circumference on frailty [16, 25]. Therefore, below-normal weight and obesity should be avoided, and optimal weight may be appropriate for older persons to maintain robustness.

In addition, the following factors were identified as contributing to the transition from robust to prefrailty: having fallen in the past year, not going out more than once a week, consulting family or friends, and feeling helpless in the last two weeks. Falls in older adults lead to fractures of the vertebrae, hip joints, and even death [26]. Furthermore, house boundedness and depressed mood were also key independent factors [27, 28]. In this study, being unable to turn to one’s family or friends for advice and feelings of helplessness in the last two weeks, including depressed mood, were found to be risk factors for the transition from robustness directly to frailty. Thus, the presence of a depressed mood may be a critical factor in preventing the transition from robustness to frailty. Marconcin et al. stated that frailty is not only a predictor of depression in older adults but also that frail participants are more likely to have depressive symptoms [24]. However, frailty is reversible. It is a dynamic state that includes improvement and progression. Indeed, in our study, 40.9% of frail individuals improved their condition to prefrailty or robustness. The finding of recovery in physical function is relevant when conducting appropriate interventions for frailty. Although some studies have evaluated every dimension of frailty, such as physical condition, house boundedness, and depressed mood, few reports have defined risk factors that consider items of KCL [22, 23, 26–28]. As a result, multiple logistic regression analysis revealed that “not going out more than once a week” was considered an independent risk factor for recovering from frailty. Therefore, going out at least once a week is recommended to prevent the transition to prefrailty and frailty and to improve housebound and depressed mood. Previous studies examined a specific factor of frailty and compared it with and without frailty [24–28]. The novelty of this study is that it examined all of the important factors using the KCL survey.

This study has several limitations. First, this study retrospectively analyzed a nationally administered questionnaire survey. The method used to select participants for the national postal KCL survey made it possible to analyze 6 years of follow-up data only for older populations. However, we reduced heterogeneity by age, which is one of the leading limitations in examining frailty trends. Second, we did not evaluate medical history, physical status, social activity, or lifestyle data. Furthermore, we did not evaluate frailty status by physical examination. Thus, this study may have included individuals who were already disabled or cognitively impaired at the baseline. However, rates of robustness, prefrailty, and frailty at baseline were comparable to previous reports [12, 15–17], suggesting that the current findings are consistent for a survey of the natural history among the community-dwelling population. Third, the reason for non-response is unknown, and it is possible that death or some other disability prevented the second survey response. Future prospective, comprehensive studies that include older adults are needed, as optimal exercise regimens, and BMI should be examined.

## Conclusion

It was found that 12.8% of the community-dwelling older adults at age 70 were frail, and at age 76, the same figure increased by 19.8%; however, this was not significant. Factors that maintained robustness were continuous walking for 15 min and a BMI of at least 18.5. Factors that transitioned robustness to prefrailty were experiencing falls in the past year and not going out at least once a week. Factors that improved frailty to robustness or prefrailty were going out at least once weekly.

## Electronic supplementary material

Below is the link to the electronic supplementary material.

Supplementary Material 1

## Data Availability

The datasets used and/or analyzed during the current study are available from the corresponding author on reasonable request.

## List of abbreviations

KCL: Kihon checklist
ADL: activities of daily living
IOR: interquartile range
BMI: body mass index
