# Supplementary material for: Determining factors that maintain physical function or increase frailty using the Kihon checklist among community-dwelling older adults: a six-year longitudinal study in Agano, Japan
Source: BMC Geriatr. 2023 May 30;23:336. doi: 10.1186/s12877-023-04055-1 (PMC10227967; doi:10.1186/s12877-023-04055-1)
Supplement: Supplementary file 1 — Supplementary Material 1 [file 12877_2023_4055_MOESM1_ESM.docx]

**Table S1**. Kihon Checklist [7]

| 1. | Do you go out by bus or train by yourself? | 0. Yes | 1. No |
| --- | --- | --- | --- |
| 2. | Do you go shopping to buy daily necessities by yourself? | 0. Yes | 1. No |
| 3. | Do you manage your own deposits and saving at the bank? | 0. Yes | 1. No |
| 4. | Do you sometimes visit your friends? | 0. Yes | 1. No |
| 5. | Do you turn to your family or friends for advice? | 0. Yes | 1. No |
| 6. | Do you normally climb stairs without using handrails or wall for support? | 0. Yes | 1. No |
| 7. | Do you normally stand up from a chair without any aids? | 0. Yes | 1. No |
| 8. | Do you normally walk continuously for 15 minutes? | 0. Yes | 1. No |
| 9. | Have you experienced a fall in the past year? | 1. Yes | 0. No |
| 10. | Do you have a fear of falling while walking? | 1. Yes | 0. No |
| 11. | Have you lost 2kg or more in the past 6 months? | 1. Yes | 0. No |
| 12. | Height: cm, weight: kg, BMI†: kg/m^2^ If BMI is less than 18.5, this item is scored | 1. Yes | 0. No |
| 13. | Do you have any difficulties eating tough foods compared to 6 months ago? | 1. Yes | 0. No |
| 14. | Have you choked on your tea or soup recently? | 1. Yes | 0. No |
| 15. | Do you often experience having a dry mouth? | 1. Yes | 0. No |
| 16. | Do you go out at least once a week? | 0. Yes | 1. No |
| 17. | Do you go out less frequently compared to last year? | 1. Yes | 0. No |
| 18. | Do your family or your friends point out your memory loss? E.g. "You always ask the same question over and over again"? | 1. Yes | 0. No |
| 19. | Do you make a call by looking up phone numbers? | 0. Yes | 1. No |
| 20. | Do you find yourself not knowing today's date? | 1. Yes | 0. No |
| 21. | In the last two weeks have you felt lack of fulfilment in your daily life? | 1. Yes | 0. No |
| 22. | In the last two weeks have you felt a lack of joy when doing the things you used to enjoy? | 1. Yes | 0. No |
| 23. | In the last two weeks have you felt difficulty in doing what you could do easily before? | 1. Yes | 0. No |
| 24. | In the last two weeks have you felt helpless? | 1. Yes | 0. No |
| 25. | In the last two weeks have you felt tired without a reason? | 1. Yes | 0. No |

^†^BMI, body mass index.
